# Supplementary figures and images for: Human umbilical cord mesenchymal stem cell-derived exosomes mitigate acute radiation-induced intestinal oxidative damage via the Nrf2/HO-1/NQO1 signaling pathway
Source: PLoS One. 2025 Jun 6;20(6):e0324238. doi: 10.1371/journal.pone.0324238 (PMC12143498; doi:10.1371/journal.pone.0324238)

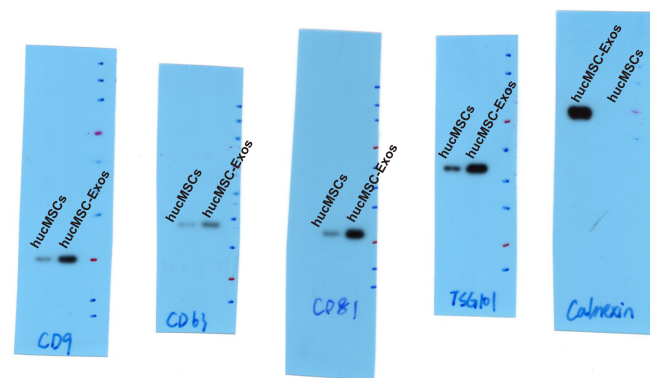

Fig. 1K . markers in hucMSC-Exos

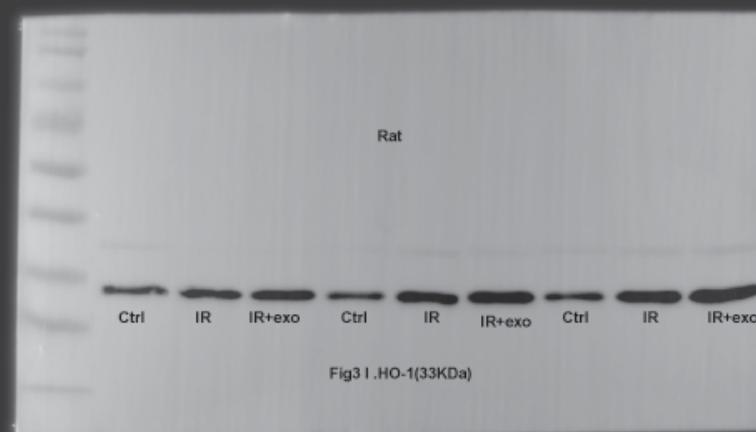

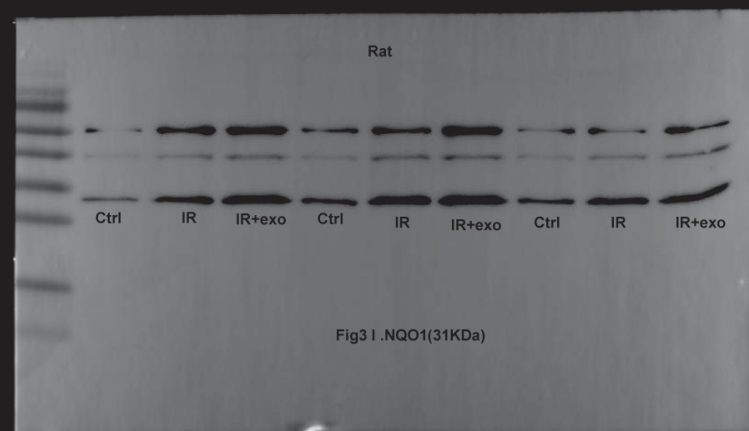

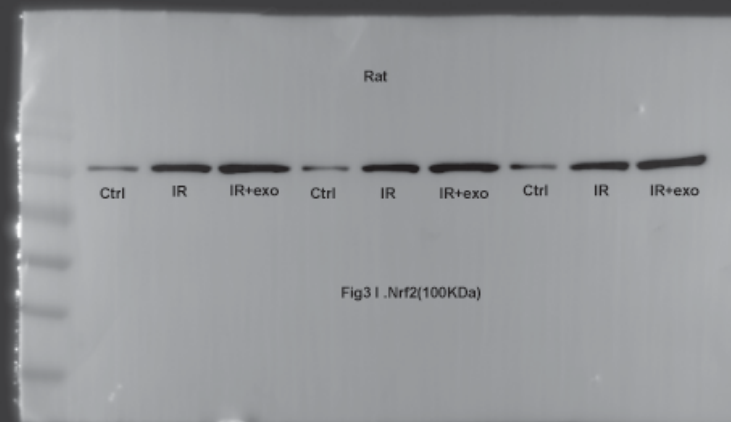

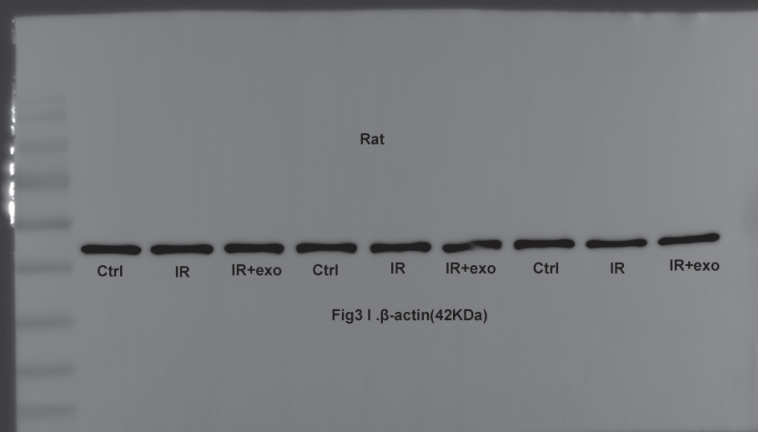

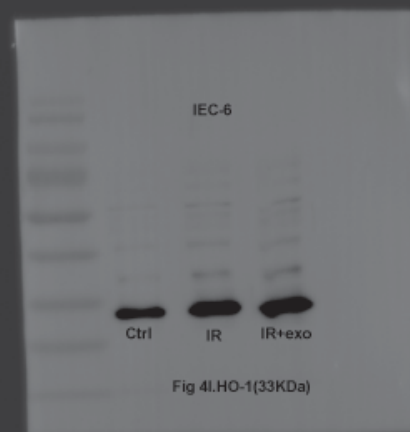

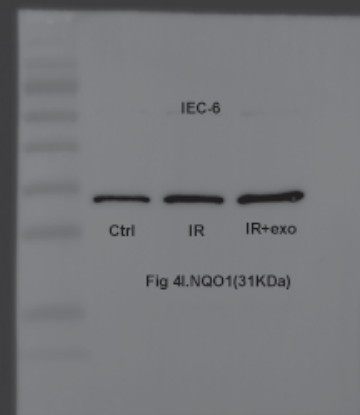

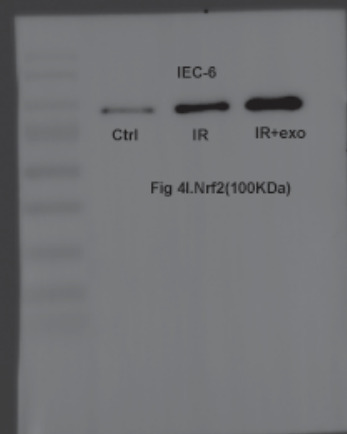

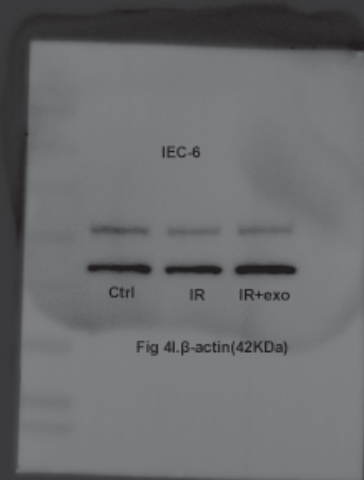

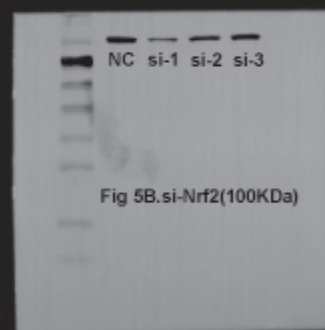

Fig 5B.si-Nrf2(100KDa)

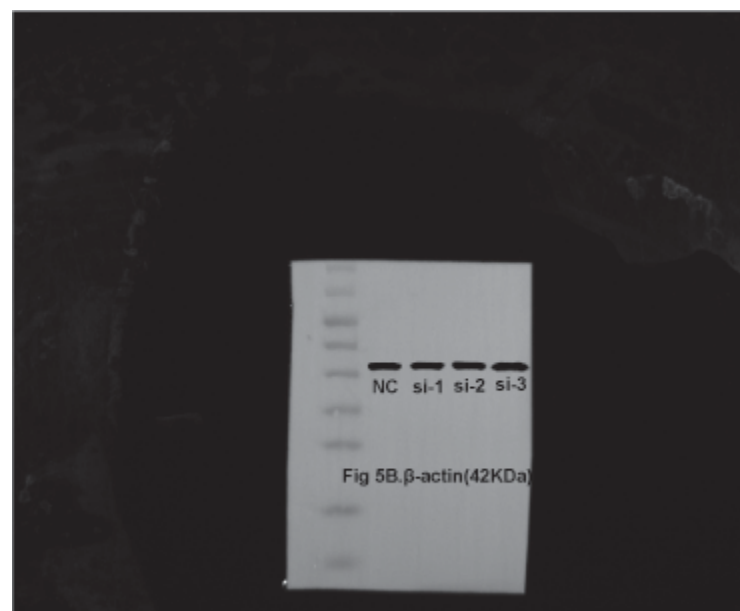

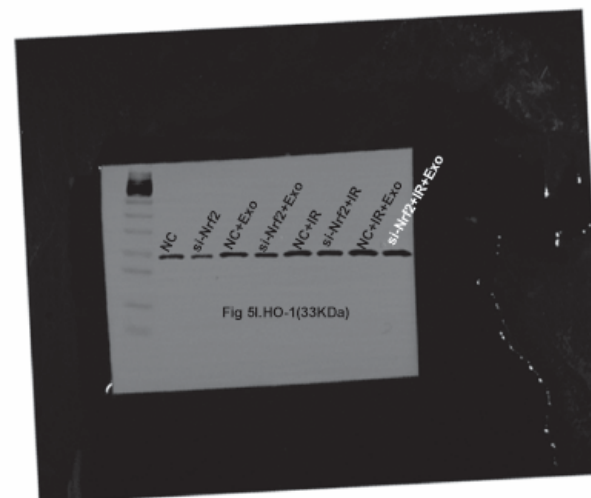

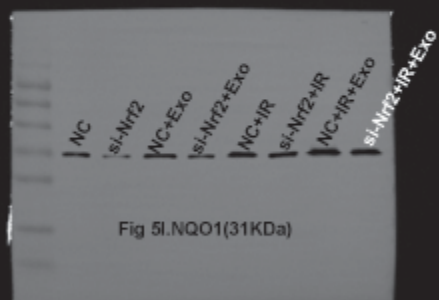

Fig 5I. NQO1(31KDa)

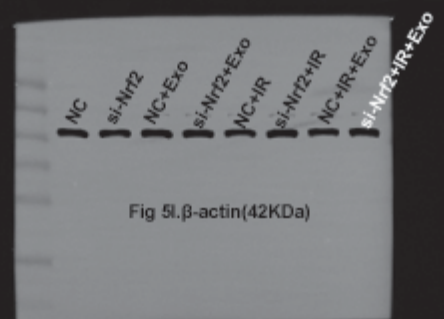

Fig 5I.  $\beta$ -actin(42KDa)

Supplement: S1 Raw images — (PDF) [file pone.0324238.s001.pdf]
