# Supplementary material for: Human umbilical cord mesenchymal stem cell-derived exosomes mitigate acute radiation-induced intestinal oxidative damage via the Nrf2/HO-1/NQO1 signaling pathway
Source: PLoS One. 2025 Jun 6;20(6):e0324238. doi: 10.1371/journal.pone.0324238 (PMC12143498; doi:10.1371/journal.pone.0324238)
Supplement: S1 Table — (DOCX) [file pone.0324238.s002.docx]

**S1 Table** PCR primers used in this study

|  | Forward primer | Reverse prime |
| --- | --- | --- |
| Nrf2 | GAGACTACCACTGTCCCCA | GAATCCTCAAAACCATGAA |
| HO-1 | ATGTCCCAGGATTTGTCCG | GGGTTCTGCTTGTTTCGCT |
| NQO1 | AGCGTCTGGAGACTGTCTG | TCTAGCTTTGATCTGGTTG |
| β-actin | TTGTAACCAACTGGGACGATATGG | GATCTTGATCTTCATGGTGCTAG |
