# Supplementary material for: Human umbilical cord mesenchymal stem cell-derived exosomes mitigate acute radiation-induced intestinal oxidative damage via the Nrf2/HO-1/NQO1 signaling pathway
Source: PLoS One. 2025 Jun 6;20(6):e0324238. doi: 10.1371/journal.pone.0324238 (PMC12143498; doi:10.1371/journal.pone.0324238)
Supplement: Attachment — Submitted filename: External revision_.pdf [file pone.0324238.s005.pdf]

Article external revision

Title:

Human umbilical cord mesenchymal stem cell-derived exosomes mitigate acute radiation-induced intestinal oxidative damage via the Nrf2/HO-1/NQO1 signaling pathway.

Hongyu Wang; Jinbao Wang; Gaosheng Yang; Yanjie Li; Weikai Chen; Jianping Yu; Xiaopeng Han

PONE-D-25-12676

A. Short description of article

Radiotherapy is one of the indispensable treatments for malignant tumors, alongside 39 surgery and chemotherapy. However, its application inevitably causes damage to 40 healthy tissues, causing in particular radiation-induced intestinal injury (RII). the mechanisms underlying ARII remain incompletely understood, and there is no established optimal treatment, with many existing therapies showing limited efficacy. Exosomes are extracellular vesicles (EVs), natural nanomaterials that can transfer specific proteins, lipids, RNA, and DNA between cells, with advantages such as low immunogenicity, high stability, ease of storage, and efficient delivery. In particular, Human Umbelical cord mesenchymal stem cell-derived exosomes (hucMSC-Exos) seams to be one of the most important candidates to treat (RII) thanks to their low immune rejection rate, tissue repair property and regeneration. This study validates the efficacy of hucMSC-Exos through in vitro and in vivo experiments and explores their potential mechanisms and pathways of action.

Strong Points of the article

The work is extremely interesting from an application point of view and in the subject matter being examined. The use of exosomes is certainly interesting and advantageous based on the points listed in the paper. In addition, the study of Nrf2 patterning is of extreme interest given its involvements with other signaling systems as well, thus paving the way for further studies

B. Weak points

Minor weak points

The text is flowing in reading, there are no inconsistencies but the form of the text should be improved. Also, space between one paragraph and the next should be added.

- **Line 158, are present 2 dots**
- **Line 126, 1x10^5 must be written $1x10^5$**
- **Line 168, 1x106 must be written $1x10^6$**
- **Line 185, which type of microscope?**
- **Line 195, which type of microscope**
- **Line 203, how did you measure the absorbance?**
- **Line 211, 1x10^5 must be written $1x10^5$**
- **Line 219, which instrument is it?**
- **The style of the references is different in the conclusion than in the rest of the article. Check that all bibliographic references are uniform with each other and adhere to the style that the journal dictates.**
- **Line 426, commas and References**
- **Images 1, 2, 3 e 4 pay attention to the scale bar. Is not visible**
- **Image 3C the words Dapi, Ros and Merge seem to refer to the image above; so bring it closer to the corresponding image.**
- **"CON" to substitute with "CTRL"**
- **Unit measures in image 4, graph B**

Major weak points

No errors or datasets were found to need revision.
