# Supplementary material for: Human umbilical cord mesenchymal stem cell-derived exosomes mitigate acute radiation-induced intestinal oxidative damage via the Nrf2/HO-1/NQO1 signaling pathway
Source: PLoS One. 2025 Jun 6;20(6):e0324238. doi: 10.1371/journal.pone.0324238 (PMC12143498; doi:10.1371/journal.pone.0324238)
Supplement: Attachment — Submitted filename: External revision_PONE-D-25-12676.docx [file pone.0324238.s004.docx]

**Article external revision**

A. **Short description of article**

**Title:** *Human umbilical cord mesenchymal stem cell-derived exosomes mitigate acute radiation-induced intestinal oxidative damage via the Nrf2/HO-1/NQO1 signaling pathway*

*Hongyu Wang^1,2^, Jinbao Wang^1,2^, Gaosheng Yang^1,2^, Yanjie Li^1,2^, Weikai Chen^2^, Jianping Yu^2**^, Xiaopeng Han^2*^*

*Manuscript number: PONE-D-25-12676*

The therapeutic value of exosomes from human umbilical cord mesenchymal stem cells (hucMSC-Exos) in the treatment of acute radiation-induced intestinal injury (ARII) is explored in this study. Through the application of both in vivo (in a rat model) and in vitro (in the IEC-6 cell line) experiments, the study discovers that hucMSC-Exos reduce oxidative stress, repair the integrity of the intestinal barrier, and promote epithelial cell proliferation. These effects are achieved through activation of the Nrf2/HO-1/NQO1 antioxidant signaling pathway. The findings classify hucMSC-Exos as a potential, cell-free treatment for avoiding radiation-induced gut damage in cancer.

B. **Strong Points of the article**

The work is extremely interesting and topical, especially in the field of acute radiation-induced intestinal (ARII) oxidative damage.

In particular is very interesting the innovative use of human umbilical cord mesenchymal stem cell-derived exosomes (hucMSC-Exos) for the treatment of ARII highlighting their ability to restore the redox homeostasis, reducing ROS and MDA, and increasing SOD and GSH-Px, using a robust in vitro and in vivo validation based on IEC-6 cells and rat models, increasing the translational relevance of the study.

Moreover it clearly identifies the involvement, as a key mediator, of the Nrf2/HO-1/NQO1 signaling pathway, in the exosome-induced protection, confirmed using siRNA to silence Nrf2, indicating the mechanistically dependence of the hucMSC-Exos protection to this pathway.

C. **Weak points**

**Minor weak points**

There are simply typos and minor adjustments to be made:

- Line 39; 40; 68; 70; 87; 132; 181; 200; 203; 207; 323; 326; 337; 340; 358; 364; 388; 459; 471; 473 there is no space after the dot;
- Line 168; 211 the number of the cells is not written with exponential, as previously done;
- Line 327; 379; 381; 404; 418; 424; 425; 426; 427; 459; 462 the citation are written at the same level of the text and not as a superscript, as previously done;
- Line 405 Ki67+ have the first letter written in lowercase and not in uppercase, as done before
- The paragraph of the Flow cytometric analysis is written with a different style respect to the other, like a laboratory protocol, I consider to rewrite this paragraph with the same style of the other;
- Line 200 what method was used to determine the protein concentration? Was a standard curve constructed first? If so, how?
- Line 202-203 what instrument was used to measure the absorbance?
- Figure 1A the scale bar is not readable and in Figure 1G and 1H the scale bar is not present;
- Figure 1K which normalizing protein was used? I would insert its image;
- Figure 4C the scale bar are not present

**Major weak points**

There are no major weak points
